# “We need to talk about it, test it, prevent it, and that is our job”: qualitative report on the awareness of primary care physicians regarding HIV in Ukraine

**DOI:** 10.3389/frhs.2024.1444575

**Published:** 2024-08-13

**Authors:** Yulia Kvasnevska, Mariia Faustova, Kseniia Voronova, Yaroslav Basarab, Yaroslava Lopatina

**Affiliations:** ^1^ AIDS Healthcare Foundation, Kyiv, Ukraine; ^2^ Department of Microbiology, Virology and Immunology, Poltava State Medical University, Poltava, Ukraine

**Keywords:** HIV, primary care, healthcare system, HIV testing, prevention of HIV

## Abstract

**Introduction:**

Approximately 240,000 people in Ukraine are living with HIV. Recent changes in medical legislation have significantly strengthened the role of family doctors and primary care pediatricians in the prevention, early detection and follow-up of patients with HIV. Thus, the purpose of the study was to examine the experience of primary care physicians in testing and providing care to patients with HIV.

**Methods:**

Qualitative semi-structured in-depth interviews with typical representatives of the target audience from different regions of Ukraine were conducted. Inductive thematic content analysis was used to analyze the data upon standardized research protocols using the Theoretical Domain System.

**Results:**

The results identify knowledge, skills, motivation, barriers, and opportunities in the context of HIV testing among family doctors in Ukraine. Primary health care providers consider HIV testing of patients to be an important part of their professional activities. In most cases, HIV testing is initiated upon detection of clinical indicators or when a person is identified as a key population. Preventive testing at the request of the patient is less common. Although most family doctors believe that they are sufficiently informed to conduct testing, there is a certain knowledge gap regarding communication with the patient and further treatment in case of a positive HIV result. The main obstacle to regular HIV testing is the reluctance and resistance of patients, as well as in some cases insufficient or non-existent tests in primary health care facilities. In order to improve the situation with HIV testing among the population, family doctors believe that it is necessary to organize regular trainings and courses to improve the skills of doctors on HIV, provide primary health care facilities with sufficient tests and establish high-quality information support.

**Conclusions:**

The obtained results clearly outline the main problems that concern family doctors in Ukraine regarding work with key vulnerable groups and persons living with HIV.

## 1 Introduction

According to UNAIDS, Ukraine ranks second in Europe in terms of HIV infection: approximately 240,000 people in the country are living with HIV (1). Despite the expansion of access to HIV testing in Ukraine, not only at the level of trust offices and AIDS centers, but also at the level of family medicine and the state program of medical guarantees, a significant number of Ukrainians still do not know their HIV status (2). Official data indicate that about 60% of key and vulnerable populations have been tested, while for the rest of Ukraine this figure is even lower (1).

For a long time, preventive medicine has been in decline in Ukraine, and the vast majority of qualified patient care has been provided at the more expensive specialized level of the healthcare system. That is why in 2016, Ukraine launched a healthcare reform, one of the priorities of which is to create a competitive, accessible, and high-quality primary healthcare system (3). To this end, the state program of medical guarantees was introduced, which regulates the amount of medical care in the main areas that a patient can receive to meet his or her needs. According to this, doctors in primary health care facilities are obliged to introduce prevention and early detection of socially dangerous diseases, conduct laboratory diagnostics of diseases, including rapid HIV tests, and identify individual risk of HIV development, if the patient needs it. It is becoming clear that recent changes in medical legislation have significantly strengthened the role of family doctors and primary care pediatricians in the prevention, early detection and follow-up of patients with HIV (3–7).

Given the above, there is no doubt that the competence and awareness of health care providers on HIV is a fundamental basis for building a quality system of care for people with HIV in Ukraine. To date, scientific databases contain numerous publications on quantitative indicators in this regard (8–11), but qualitative research results are limited. However, such data focuses on understanding experiences, attitudes and beliefs to identify critical patterns that cannot be quantified.

## 2 Methods

Thus, the purpose of the study was to examine the experience of primary care physicians in testing and providing care to patients with HIV.

### 2.1 Research design

**Figure 1:**
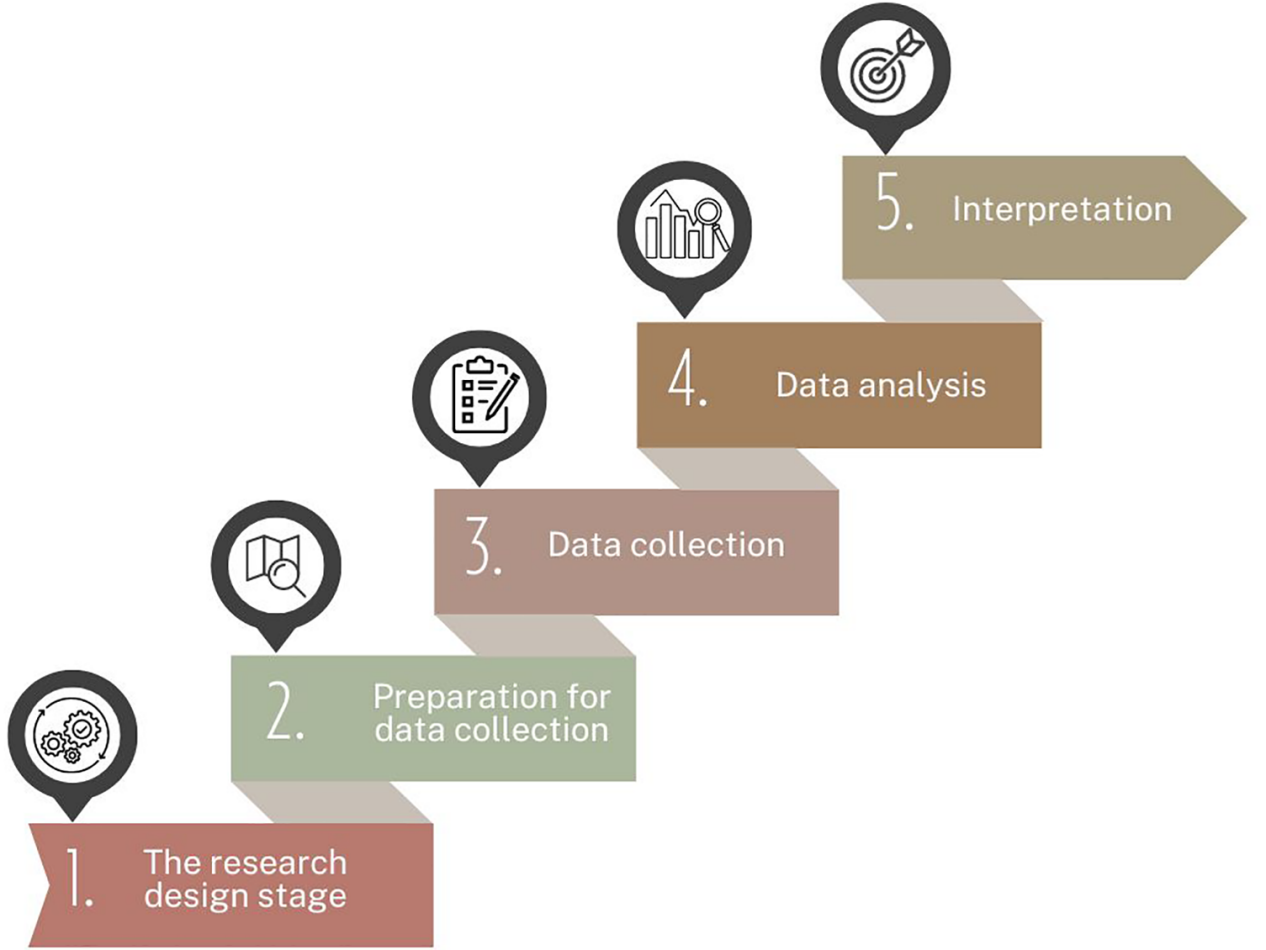
The flowchart of study process.

Qualitative semi-structured in-depth interviews with typical representatives of the target audience were conducted in July-August 2023 (Figure 1).

Respondents from six regions of Ukraine took part in the survey: Kyiv, Vinnytsia, Dnipropetrovs'k, Kirovohrad, Lviv, and Odesa regions (Table 1). The regions included in the study cover the central, southeastern, southern, and western parts of the country.

**Table 1:** Geographical boundaries of the study.

No	Region	Exclusions	Part of the territory of Ukraine
1	Kyiv	Kyiv	Central
2	Vinnytsia	Vinnytsia	Central
3	Dnipropetrovska	Dnipro, Kryvyi Rih	Southeastern
4	Kirovograd	–	Central
5	Lviv	Lviv	Western
6	Odesa	Odesa	Southern

Primary healthcare facilities in small towns and rural areas were selected for interviews. Given the developed networks of healthcare facilities in large settlements, cities with a population of more than 500,000 inhabitants were excluded from the study to ensure quality data.

Primary health care facilities were invited to participate in the study by email and phone. If an institution refused, the next one was selected from a randomized list until the required number was reached.

### 2.2 Survey participants

Twenty six healthcare professionals from primary healthcare facilities, at least 4 representatives from each oblast, took part in the survey. Respondents' eligibility for the study was determined by the following inclusion criteria: qualification as a family doctor, pediatrician, or general practitioner and at least three years of professional experience with providing services for HIV testing. Medical staff who did not meet the above criteria or were not willing to participate in the interview were not included in the study.

### 2.3 Data collection

The project team developed, refined, and agreed upon standardized research protocols using the Theoretical Domain System (TDS) to identify knowledge, skills, motivation, barriers, and opportunities in the context of HIV testing (12, 13).

Semi-structured in-depth interviews were conducted privately in an isolated, locked room at the respondents' place of work. Interviewers were trained in qualitative research methods and interviewing rules, provided with detailed instructions, and received an interview guide and protocol developed by the research team before the study began. Respondents and interviewers did not know each other beforehand. The healthcare workers who participated in the survey were familiarized with the purpose and objectives of the interview, and had full information about the organization that initiated the study. All partisipants had signed an informed consents before the start of the research. The study was approved by the Bioethics Commission of Poltava State Medical University, Poltava, Ukraine (#210, 23.11.22). The data obtained was recorded verbatim and encrypted to preserve anonymity.

The communication lasted about 1 h and 10 min and included 34 open-ended questions about the respondents' experience, knowledge and skills in the context of HIV testing, regularity of such testing, level of awareness, experience of referral and cooperation with health care facilities providing care to HIV patients, motivation, barriers and obstacles, as well as ways to improve HIV testing among family doctors.

### 2.4 Data analysis

Inductive thematic content analysis was used to analyze the data. Based on the transcribed interviews, the main thoughts of the respondents were identified and coded with NVivo 12.2 Software (QSR) by two independent coders (YuK, MF) to systematize the results. Repeated patterns of codes were grouped together to form generalized themes.

## 3 Results

Based on the results of the interviews with primary care physicians, a number of themes were identified within the main domains (Supplementary Table S1). Topics that were less frequently encountered were analyzed and included in the summary table.

It was found that 100% of the surveyed health care workers, regardless of their category and work experience, had previously interacted with patients who had indications for testing or confirmed HIV infection. In general, primary health care providers perform HIV testing on average 1–2 times a week. Thus, in their responses, respondents indicated that HIV testing is not mandatory and therefore is prescribed no more than once a month. At the same time, there were also doctors whose practice required such testing almost daily. This implies that the number of HIV tests in primary health care facilities can vary from 1 to 20 tests per month, depending on a number of factors.

Respondents pointed out that the frequency of HIV testing depends on the settlement: rural residents are much less likely to seek testing. This pattern is primarily due to the prevalence of elderly people among the patient population. According to the respondents: “Older people are skeptical about the need for HIV testing and very rarely visit a family doctor for this purpose” (P 5) In addition, in small settlements, people usually know each other well, so primary care physicians do not offer such testing to people who do not belong to key and vulnerable populations without clinical indications, and patients do not seek help on their own because of fear of disclosure.

Also, among the factors affecting the frequency of HIV testing in the last two years, primary care physicians often noted an increase in the number of medical examinations for military enlistment offices and temporarily displaced persons amid the full-scale war in Ukraine.

Analyzing the results of interviews with primary care physicians, we can identify typical cases in which they recommend/conduct HIV testing:

- The patient belongs to key and vulnerable populations
- Patient is often ill
- Unexplained deterioration of the patient's condition with an unclear diagnosis
- Before surgical intervention
- If official confirmation of HIV absence is required (medical examination for employment, guardianship, military registration and enlistment office, etc.)
- At the patient's own request

At the same time, respondents indicated that patients rarely seek HIV testing of their own free will, and mostly at the trust office.

An interesting fact was the frequent use of the words “persuade” and “ask” when describing the process of obtaining patients' consent for HIV testing.

As part of the survey, doctors were asked to express their confidence in the level of knowledge about identifying, testing and counseling HIV-positive patients. The vast majority indicated “mediocre knowledge” and “feeling of uncertainty”. However, in general, all respondents assessed their personal awareness of the above issues as sufficient to perform their work, as they consider themselves an “intermediate link” between patients and AIDS centers and/or infectious disease specialists and are not directly involved in treatment. It is worth noting that the vast majority of surveyed doctors expressed a desire to improve their knowledge about HIV, namely

- identification of key and vulnerable groups;
- peculiarities of working with pregnant women and other sensitive populations;
- soft skills: communication with patients on the topic of HIV diagnosis, finding an individual approach, etc;
- how test systems work;
- further treatment process, medications, their regimens and dosages.

All respondents indicated that they had at least once attended training, courses or workshops on HIV during their practice, but it was rarely done on a regular basis. At present, the vast majority of doctors undergo advanced training on HIV on average no more than once a year. The analysis of the respondents' answers showed that the COVID-19 (COronaVIrus Disease of 2019) pandemic with the transition to online learning, and later the war, had a negative impact on the regularity and quality of acquiring new knowledge and skills in testing and interacting with HIV-infected patients. Moreover, some doctors among the respondents had difficulty recalling the topics and approximate time of the last in-service training on these issues, indicating the prevalence of self-education via the Internet and communication with experienced colleagues. However, a significant number of study participants positively assessed the experience of cooperation with non-governmental organizatios that had previously conducted HIV prevention training and indicated the need for regular repetition.

The study found that the vast majority of primary health care providers have the necessary regulatory documents on HIV counseling, testing and treatment at their workplace. Others said they knew where they were and could find them if necessary. However, the vast majority of respondents are familiar with the main orders of the Ministry of Health and protocols governing prevention, testing and interaction with people who test positive for HIV. At the same time, it is worth noting that almost all of them did not remember the exact numbers of these regulatory documents and indicated that they did not follow the regularity of their updates and additions. A natural and frequent response was the desire to expand their knowledge of the legal framework for working with HIV-infected people and to have guides/checklists, etc. for quick step-by-step responses to certain HIV-related situations. An analysis of respondents' answers about the available protocols, orders and other regulatory documents that they use in their practice showed that such documents are mostly more formal and do not have a practical impact on the work of family doctors. The doctors themselves explain this by lack of time, as they have certain limitations on seeing one patient, frequent and easier use of alternative information sources, and the prejudice that family doctors only provide primary HIV testing.

Most of the health facilities surveyed had rapid HIV diagnostic tests. Few knew the details of the manufacturer and the principle of operation of such a test, most often it was described as “similar to a covid test”. A nurse, either on her own or under the supervision of a doctor, most often performs the test, while a doctor himself or herself does it much less often.

The results of the interviews show that primary care physicians determine the need for HIV testing during a conversation with the patient, finding out about the patient's lifestyle, previous laboratory tests, complaints, and comorbidities. This allows us to determine whether the patient belongs to key and vulnerable populations or suspects infection, as the vast majority of patients are offered mandatory HIV testing (Table 2). At the same time, there were some doctors who usually avoided suggesting preventive testing because they did not know the right approach and were afraid of the patient's reaction.

**Table 2:** The main indicators that primary care physicians are guided by when recommending HIV testing.

By categories of persons	By clinical signs
Belonging to socially disadvantaged Categories of the population	Prolonged fever
Pregnancy	Changes in blood test results
Injecting drug use	Diarrhea
Presence of tuberculosis, hepatitis B and C	Frequent colds
Marriage or partnership with an HIV-infected person	Prolonged poor health, weakness
Presence of tattoos, piercings, manicures	Skin rashes, pustules
	Rapid weight loss, loss of appetite
	Difficulty in determining symptoms and diagnosis

Doctors noted frequent cases of patients refusing to undergo HIV testing primarily due to fear of getting a positive result or concealing an existing disease, as well as uncertainty about the confidentiality of the information received. In such cases, all respondents understood the need to find an individualized approach to the patient and to find the right reasoned explanations. Nevertheless, according to the respondents, quite a few patients still do not accept the doctor's recommendations. That is why doctors repeatedly emphasized the need to use “soft skills” during the interviews: to calm, support, find an individual approach, create comfortable conditions for consultation without unauthorized persons, and follow up to see if the patient will seek further treatment.

The main obstacle to regular HIV testing of patients was primarily identified by all interviewed health care workers as the fear of disclosure in case of a positive result. Of course, 100% of the respondents noted their strict adherence to confidentiality, the main ones being keeping separate registers for HIV-infected people, encrypting information about the diagnosis, etc. However, the overwhelming majority of respondents believed that the main factor contributing to the patient's consent to testing was the formation of trust in doctors.

Usually, primary care physicians conduct repeated testing or, if necessary, refer patients for additional HIV testing if they receive an initial positive result. Analyzing the interview data, we can trace a generalized algorithm of actions for primary care physicians when HIV is detected. It consists of informing the patient of the test result, calmly and carefully informing them about the course, characteristics of the disease, and further actions, followed by referral to a trust office or infectious disease specialist. All respondents characterized the process of interaction and referral of patients to doctors directly involved in HIV treatment as “well-established” or “very good”.

According to the results of the interviews, primary care physicians defined their place in the HIV care system as the first step for patients. The respondents considered their main tasks to be primarily informing patients about the ways of infection, identifying potential risks, timely HIV diagnosis, and explaining the algorithm of actions in case of a positive result. The respondents themselves had the following to say about this: “We need to talk about it, test it, prevent it, and this is our job” (P 20), “I believe that every doctor in Ukraine has a place in this prevention” (P 4).

According to the study, the factors that motivate family doctors to conduct HIV testing are primarily personal successes in the work of doctors in this regard. Most often, respondents said that the best incentive for further active work on these issues is awareness of their role in timely detection of HIV and proper referral of patients who have received a positive result. Also, doctors indicated that understanding the problem of HIV prevalence in Ukraine and the desire to provide assistance to all those who need it are direct motivators for active work on HIV diagnostics and testing. At the same time, there was also a part of respondents who considered the perception of HIV testing as a regular mandatory part of their job duties, the mere availability of tests and equipment in the healthcare facility to use, and financial bonuses to be the main factors that motivated them to work.

The main difficulty with HIV testing in primary health care facilities, according to the surveyed doctors, is the reluctance and sometimes resistance of patients for various reasons. These include fear of confidentiality violation, age, lifestyle, lack of trust in medical staff, and, most importantly, low public awareness of HIV, its consequences, and the importance of timely detection. These responses suggested further factors that hinder testing efforts, such as a lack of patient information materials, especially in rural areas, and a lack of a testing culture among the population. In addition, respondents identified the lack of tests in healthcare facilities and the recent decline in the relevance of HIV amid the COVID-19 pandemic and the war in Ukraine as barriers.

Among the resources that are lacking for quality HIV testing, primary care physicians most often pointed to a lack of staff, time, tests, knowledge of new approaches to working with potential and risk groups among physicians themselves, and patient awareness.

It was extremely important to hear the opinions of primary care practitioners on the possibility of improving the quality of diagnosis and care for HIV patients. They see the prospect of educational work. The overwhelming majority of responses to this question concerned strengthening work with children and young people by involving them in cultural events, lectures, discussions, as well as creating and popularizing videos and cartoons about HIV. Repeatedly during the interviews, doctors emphasized the need to increase the number of visual materials: wall posters, stands, brochures, booklets, etc. with information on HIV transmission, testing, and step-by-step instructions for action in case of suspected HIV infection for distribution among the population. As the doctors noted: “We notice that if you hang it on the walls, they (patients) sit in lines and read it” (P 11). In addition, respondents suggested that social media could be used to promote contraception and create a culture of preventive testing. In general, primary care physicians considered it necessary to transform doctor-patient communication into a more informative type of communication.

Primary care physicians considered increasing the frequency of practice-oriented training for both doctors and nurses who are also involved in working with this category of people to be an important factor in improving the provision of medical care to patients with HIV. Doctors emphasized the need for closer direct contact with specialists from Trust Centers and organizations dealing with HIV issues, who could, for example, sometimes attend appointments with a doctor in case of need for additional counseling of patients, etc.

## 4 Discussion

Analyzing the data from the interviews, it is worth noting the homogeneity of the topics identified within the TDS domains. These results demonstrate that regardless of geographic location, work experience, age, and other factors, primary care physicians have a similar vision of the situation with HIV testing and work with key and vulnerable populations, and face the same concerns and problems. It is worth noting the frequent overlap of answers to certain questions and sometimes even the use of similar words and phrases to describe their own experience.

When conducting HIV testing and treating infected patients, it is very important that the doctor has comprehensive information and is trained in who should be tested, how and how to follow up on a patient with a positive result in the future. After all, it is obvious that a lack of clinical knowledge jeopardizes success in the work (14, 15). In addition, in-depth qualitative research among patients themselves confirms the importance of the initial experience of interaction with a doctor, which forms the patient's confidence, openness and interest in continuing treatment in the future (16). This qualitative study demonstrates a clear understanding of the importance of their role in HIV testing among primary care physicians. Such results, obtained by us, coincide with previous studies in Moldova, a neighbor of Ukraine, where, according to the results of the survey, doctors have a clear awareness and belief in the importance of their role in the path of early detection and treatment of HIV (17). However, the vast majority of Ukrainian family doctors are aware of the lack of knowledge and skills to implement mechanisms for quality care in case of suspected or diagnosed HIV. Along with this, Rogowska-Szadkowska D. and others in their research indicate a similar situation in Poland. According to their data, about a third of family doctors note a lack of knowledge about treatment, prevention and principles of early diagnosis of HIV (18). It is worth noting that, according to the literature, countries such as the UK, Switzerland, and Germany have a lower level of late diagnosis of HIV, which is often directly related to the presence of clear algorithms for actions in case of suspicion for primary care medical providers (18–20).

It was worrying that the vast majority of respondents saw the need to improve their knowledge of HIV, but most often resorted to self-education and searching for information on the Internet.

A significant problem revealed by this study is the low level of soft skills among doctors, which respondents repeatedly emphasized the need to improve. After all, patients who test positive for HIV often experience psychological distress, anxiety, and panic due to lack of awareness of the further changes in their lives that are associated with it (21, 22). And it is the primary care physicians who are the first to interact with this category of people who should be aware of this and have the skills to reduce patient anxiety and build trusting long-term relationships. Moreover, the lack of the right approach to patients and the ability to establish a friendly dialog is directly related to the low level of HIV testing among the population of small towns and villages in Ukraine.

The results of the interviews show that a lack of awareness about HIV among both doctors and patients can lead to stigmatization, fear of testing, and lack of preventive measures. People who are not well informed about the benefits of testing and the possible consequences of a positive diagnosis may avoid it, which in turn increases the risk of HIV transmission.

## 5 Conclusions

Primary health care providers consider HIV testing of patients to be an important part of their professional activities. Despite this, the frequency of testing varies significantly, depending on the needs and willingness of patients, the presence of key and vulnerable populations, clinical indicators, type of settlement, and age of the patient.

In most cases, HIV testing is initiated upon detection of clinical indicators or when a person is identified as a key population. Preventive testing at the request of the patient is less common.

Although most family doctors believe that they are sufficiently informed to conduct testing, there is a certain knowledge gap regarding communication with the patient and further treatment in case of a positive HIV result.

The main obstacle to regular HIV testing is the reluctance and resistance of patients, as well as in some cases insufficient or non-existent tests in primary health care facilities.

In order to improve the situation with HIV testing among the population, family doctors believe that it is necessary to organize regular trainings and courses to improve the skills of doctors on HIV, provide primary health care facilities with sufficient tests and establish high-quality information support.

The obtained results clearly outline the main problems that concern family doctors in Ukraine regarding work with key vulnerable groups and persons living with HIV. Systematization of such gaps in the work of doctors, awareness of their barriers and experiences can become the basis for revising educational programs for medical students or improving the qualifications of specialists, as well as strengthening the work of organizations interested in this in order to increase the level of awareness of HIV-related medical service providers in Ukraine in the future.

## 6 Study limitations

1. The research was conducted in a limited circle of medical institutions of Ukraine, which could be expanded in the future.
2. The research is exclusively qualitative, although some data would be interesting to be further characterized by quantitative indicators on larger data samples.
3. The study was conducted in Ukraine during a full-scale war, which could have influenced the opinion of the respondent doctors.

## Data Availability

The raw data supporting the conclusions of this article will be made available by the authors, without undue reservation.
