# Supplementary material for: “We need to talk about it, test it, prevent it, and that is our job”: qualitative report on the awareness of primary care physicians regarding HIV in Ukraine
Source: Front Health Serv. 2024 Aug 13;4:1444575. doi: 10.3389/frhs.2024.1444575 (PMC11347399; doi:10.3389/frhs.2024.1444575)
Supplement: Supplementary file 1 [file Table1.docx]

Supplementary Table 1

Summary of the main topics, according to TDS domains

| TDS domain | Topics | Key quotes |
| --- | --- | --- |
| Knowledge | Prevention of HIV infection  HIV testing  Qualification level for HIV testing  Training on HIV issues  Principles of testing systems work  Further treatment process, medications, their regimens and dosages | *"...We encounter people every day, we have been offering them testing since the relevant orders appeared..."* (P 21)*, "In terms of HIV diagnostics, I cannot say that we have a very broad scope of work."* (P 9)*, "We test as needed: when they need to be hospitalized or undergo a special medical examination, where they also need it. We test exactly those patients who need it"* (P 3)*, "...I have no training in this regard, no special certificates, no. Only my own desire"* (P 11) |
| Skills | Knowledge of the documentary regulatory framework on HIV  Ability to conduct HIV testing  Skills to determine indications for HIV testing  Skills of counseling and interaction with a patient who has received a positive HIV result  Maintaining confidentiality in HIV testing  Soft skills | *"For example, I understand that even the very arrival of a patient to a healthcare facility is already a medical secret"* (P 10)*, "...we fill out all the documentation, information agreement, where all this is discussed, spelled out, the patient fills it out by hand, reads it... He knows that this is all our common secret, which remains only in this paper form"* (P 6)*, "...we need to look for ways, if a person is worried, we need to calm him down, we need to say that this is not a sentence, modern medicine allows to overcome this problem and together we can achieve the goal"* (P 4). |
| Motivation | Personal importance in HIV prevention  Incentives for HIV testing | *"I believe that this is a part of my job. I believe that it is not something so ephemeral... We need to talk about it, test it, prevent it, and that is our job."* (P 20)*, "I don't know, I don't need motivation in principle"* (P 9)*, "...you want to get to the bottom of it, you know, when you are a doctor, a patient comes to you and you examine him, talk to him, take anamnesis, you want to get to the bottom of it..."* (P 1) |
| Barriers | Difficulties in providing HIV testing services  Lack of resources | *"We have very few tests, very few people come. The obstacle is that they don't want to."* (P 6)*, "Not the patient's desire, categorical unwillingness"* (P 4)*, "Maybe confidentiality, because people in the village are very scrupulous about this, they worry that it will not be possible to maintain this confidentiality"* (P 14) |
| Opportunities | Improving the quality of HIV diagnosis and testing  Interaction with healthcare professionals who provide care to people living with HIV | *"It is necessary to make children's videos, cartoons, from the age of 10, accessible to convey this message."* (P 17), *"It is necessary to conduct some kind of training, to bring doctors and nurses together, because it is like a doctor and a nurse working in a team."* (P 2), *"Gather all family members or even heads of departments, explain the work."* (P 20). |
